# Resilin microjoints: a smart design strategy to avoid failure in dragonfly wings

**DOI:** 10.1038/srep39039

**Published:** 2016-12-14

**Authors:** H. Rajabi, A. Shafiei, A. Darvizeh, S. N. Gorb

**Affiliations:** 1 Institute of Zoology, Functional Morphology and Biomechanics, Christian-Albrechts-University, Kiel, Germany; 2 Department of Mechanical Engineering, University of Guilan, Rasht, Iran; 3 Department of Mechanical Engineering, Anzali Branch, Islamic Azad University, Bandar Anzali, Iran

## Abstract

Dragonflies are fast and manoeuvrable fliers and this ability is reflected in their unique wing morphology. Due to the specific lightweight structure, with the crossing veins joined by rubber-like resilin patches, wings possess strong deformability but can resist high forces and large deformations during aerial collisions. The computational results demonstrate the strong influence of resilin-containing vein joints on the stress distribution within the wing. The presence of flexible resilin in the contact region of the veins prevents excessive bending of the cross veins and significantly reduces the stress concentration in the joint.

The wings of Odonata may undergo extremely large deformations. The deformations applied to the wings are not only due to aerodynamic and inertial forces in flight^1^,^2^,^3^,^4^, but also because of the interactions between wings and solid obstacles. Such physical contacts may take place several times in the insects’ daily activities such as taking off, mating, territory defence and predation. Video S1 shows a single pair (*Sympetrum sanguineum*) in tandem. Several contacts between the male’s body and female’s wings can be observed when the male is trying to grip the female by the head. The imposed mechanical forces and deformations may result in unexpected stress concentrations especially in the regions where veins cross each other. How do odonate wings avoid or minimize potential damage caused by high localized stresses?

The answer to this question probably lies in the specific deformability of the wings. Although several parameters influence the deformation of the wings in Odonata, considering their distribution over the wing, vein microjoints have been recognized as one of the most effective elements, in contrast to the others^5^,^6^. Recent investigations by means of different microscopy techniques have confirmed the occurrence of the resilin in the vein joints of numerous dragonflies^7^,^8^,^9^,^10^ and other insect species^11^,^12^. We have shown numerically that the vein microjoints strongly contribute to the camber formation in flight by increasing the torsional flexibility of the wing^5^. Hence, it is plausible to hypothesize that the microjoints may have an additional functional significance by avoiding the wing failure under impact.

There are several types of vein joints with different geometries and material compositions in the wings of Odonata^8^. However, in the general case, they all consist of three main components: (i) two veins which directly or indirectly connected to each other, (ii) a resilin patch which is located in the interval between the veins when they are not in direct contact, and (iii) a spike on one side or two spikes on both sides (dorsal and ventral) of one of the veins. Figure 1A shows two vein joints located on the right- and left-hand sides of a longitudinal vein of the dragonfly *S. vulgatum*. The joint located on the left-hand side of the figure contains all the mentioned components, while that located on the other side has neither a spike nor a resilin patch.

The cross section of a typical vein joint in the wings of the damselfly *Matrona basilaris basilaris* is illustrated in Fig. 1B. The light microscopy image shows a longitudinal vein which is connected to a spike-containing cross vein on the right-hand side. This section is stained with toluidine blue suggesting the presence of the resilin in the regions with the sapphire to bright blue colour. As seen in the figure, the resilin is mainly localized at the contact area between the veins and also in the endocuticle layer of the longitudinal vein. Considering the presence of melanin, the resilin-dominated endocuticle, which is the innermost layer in the longitudinal vein microstructure, is stained violet^13^. The unsclerotized cuticle in this section has remained unstained.

We used a simple two-dimensional (2D) FE model to simulate the mechanical behaviour of the vein joint shown in Fig. 1B, subjected to a force applied to the dorsal and ventral sides of the cross vein (Fig. 2A,B). Although the longitudinal veins in odonate wings have a more complex composite structure consisting of several cuticular layers^13^,^14^, we simplified the longitudinal vein in our model by considering two main layers of “hard” and “soft” cuticles (see materials and methods in the supplementary materials). This model serves as the main model in our study and we refer to it as Model 1 throughout the manuscript.

Figure 2A,B illustrates four consecutive deformation steps of the model subjected to a point force applied to the free end of the cross vein. As seen in this figure, the model undergoes nearly the same deformation under both dorsal and ventral loadings before the occurrence of a physical contact between the spike and the adjacent longitudinal vein. When the contact takes place, the spike on the dorsal side of the cross vein prevents its further rotation about the axis of the longitudinal vein. However, this is not the case for the rotation of the cross vein in the other direction and the joint can be deformed without limit.

This simulation shows the dorso-ventral asymmetric deformation of the joint, which potentially can result in the asymmetry of the wing deformation subjected to forces applied to its dorsal and ventral sides. Such an asymmetry has already been reported for various insect species^15^,^16^,^17^. The asymmetric deformation of the wing has an important influence on the insect flight, since it facilitates camber formation, and therefore the aerodynamic lift production when the wing is loaded from the ventral side^17^. The wing camber and the basal complex were known to be two sources of such an asymmetry^16^,^17^. However, the result of the current study suggests that the joint-associated spikes may be another reason for the anisotropic dorso-ventral stiffness of the wing.

In order to understand the mechanical role of the resilin patch, we developed another FE model (Model 2) by replacing the soft cuticle and the resilin patch in the first model with a layer of the hard cuticle (Fig. 2C). Although the decrease in the joint deformation was predictable, we did not expect such an infinitesimal deformation under the same loading condition. The cross vein in this model shows a rotation which is almost 170 times less than that of the main model.

The selected screenshots in Fig. 2A–C also represent the evolution of the elastic strain energy density stored in the deformed models. As seen here, the strain energy density in Model 1 is mainly localized in the flexible resilin patch, whereas the strain energy density in Model 2 is somewhat distributed throughout the joint and the cross-vein. Videos S2,S3,S4 show the deformation process and the evolution of the elastic strain energy density of the models illustrated in Fig. 2A–C, respectively.

The high strain energy density stored in the contact area between the veins in Model 1 (Fig. 2A–C) suggests a more significant role of the resilin patch in the deformability of the joints, compared to that of the soft endocuticle in longitudinal veins. Hence, it is expected that the compliant core in the microstructure of the longitudinal vein mainly contributes to the mechanical behaviour of the vein itself^14^ rather than the rotation of the cross-vein about the axis of the longitudinal vein. On the other hand, large deformations may negatively impact the load-carrying capacity of the wing. Therefore, the stiff cuticular spike located on the joint prevents such a large deformation by establishing a physical contact with the adjacent vein. The association of spikes with resilin-containing vein joints in most of the observations^8^ further supports this conclusion.

We see that applying the same force to the developed models results in only a small deformation of Model 2, compared to the large deformation of Model 1. Therefore, considering the limitations of such a comparative study, we decided to apply the same displacement to both models, simulating the mechanical behaviour of the joint in contact with an obstacle. Similar to the previous loading condition, the deformation was applied to the free end of the cross vein (Fig. 2D,E).

The representative screenshots showing the displacement of the models subjected to a mechanical deformation on the ventral side of the cross vein are given in Fig. 2D,E. Comparison of the deformation patterns of the models suggests a considerable difference. The cross vein in Model 1, containing the soft endocuticle and the resilin patch, undergoes a large angular deformation about the axis of the longitudinal vein. In contrast, the relatively small rotation of the cross vein about the longitudinal vein in Model 2 is associated with the large bending deformation of the cross vein.

Figure 2D,E also represents the distribution of the maximum principal stress within the deformed models. The increase in the magnitude of the deformation in each step remarkably increases the localized stress at the contact area of the veins in Model 2. In contrast, the vein joint in Model 1 shows only a small stress concentration in this region. The magnitude of the maximum stress in Model 2 is almost 80 times higher than that of Model 1. Videos S5 and S6 show the evolution of the stress in Model 1 and Model 2 by the increase of the applied ventral displacement, respectively.

The stress concentration in the model without resilin patch (Model 2) is much larger than the average value of the stress in this model. This highly localized stress due to the use of the stiff material between the veins may cause failure of the joint in this contact zone. Considering that such an irreversible damage in the wing may drastically affect the flight performance of the insect, we can suggest that the presence of low-modulus resilin patches in the vein joints is a “smart” design strategy to avoid the wing failure in Odonata. The use of similar design mechanisms may improve the durability and performance of artificial micro-air vehicles (MAVs) with flapping wings.

The use of a 2D modelling technique enabled us to include the detailed microstructure of a vein joint into our model and to decrease the extensive computational costs resulting from the use of a complex three-dimensional (3D) model. The models developed in this article allowed us to conduct a qualitative comparative study of the influence of the resilin-dominated region in the joint structure on its mechanical behaviour. Future studies may focus on detailed 3D modelling of vein joints from different wing parts, considering their different dimensions, geometries and material distributions. Such models can be utilized to obtain quantitative data on local mechanical behaviour of dragonfly wings.

## Methods

### Finite element modelling

**Figure 1:**
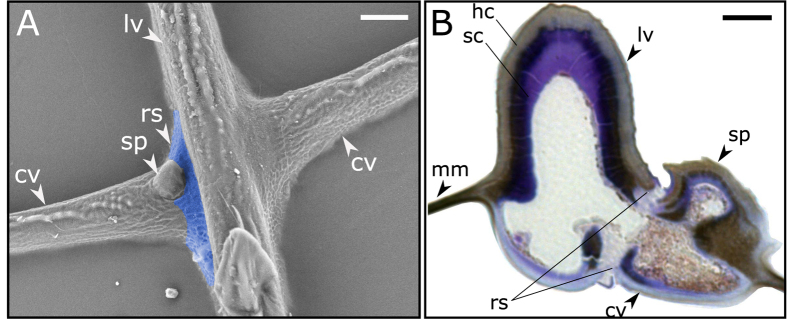
(**A**) Scanning electron micrograph of two cross veins connected to a longitudinal vein in the forewing of *S. vulgatum*. The vein joint located on the left-hand side of the figure contains a resilin patch (marked blue) at the contact area of the veins and a spike located on the cross vein. The joint on the right-hand side of the longitudinal vein has neither a spike nor a resilin patch. (**B**) Cross section of a vein joint of *M. basilaris basilaris* stained with toluidine blue (reconstructed with permission from ref. 13). The presence of resilin is illustrated by the sapphire or bright blue colour at the contact area of the longitudinal and cross veins and by the violet colour in the soft endocuticle. The hard cuticle has remained unstained, but due to sclerotization appears grey-brownish. lv, longitudinal vein; cv, cross vein; mm, membrane; rs, resilin; sp, spike; hc, hard cuticle; sc, soft cuticle. Scale bars: 10 μm.

**Figure 2:**
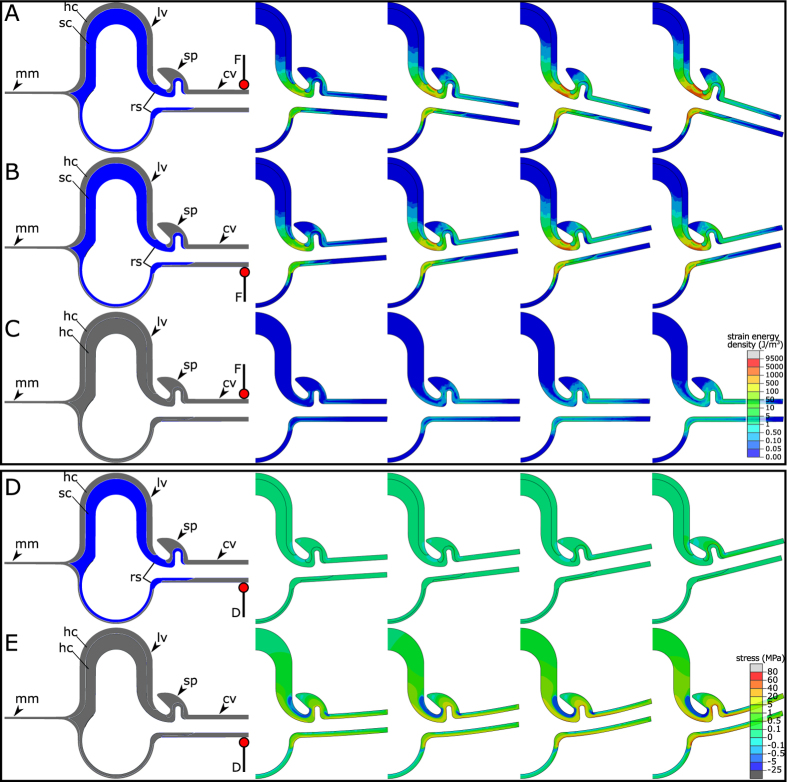
(**A**–**C**) Representative screenshots of the deformation pattern of (**A**) Model 1 subjected to a force on the dorsal side of the cross vein, (**B**) Model 1 subjected to a force on the ventral side of the cross vein, and (**C**) Model 2 subjected to a force on the dorsal side of the cross vein, indicating the considerably larger deformation of Model 1, compared to Model 2. The distribution of the elastic strain energy density is shown in the deformed shapes of the models. In Model 1, the strain energy density is concentrated in the contact zone of the veins, suggesting the role of the resilin patch in the deformability of the joint. (**D**,**E**) Representative screenshots of the deformation pattern of (**D**) Model 1 and (**E**) Model 2 subjected to a displacement applied to the ventral side of the cross vein. The figure shows the rotation of the cross vein about the axis of the longitudinal vein in Model 1, but the bending of the cross vein in Model 2. The distribution of the maximum principal stress within the models indicates a high stress concentration in Model 2 due to the removal of the resilin patch and the soft cuticle. lv, longitudinal vein; cv, cross vein; mm, membrane; rs, resilin; sp, spike; hc, hard cuticle; sc, soft cuticle; F, external force; D, applied displacement.

The data from our previous microscopy studies^8^,^9^ were used here to develop numerical models of the vein joints in Odonata. The finite element (FE) software package, ABAQUS/Standard (v6.14), was utilized for this purpose. The developed models are two-dimensional (2D), consisting of a longitudinal vein which is connected to a cross vein and a spike located on the cross vein. The longitudinal vein in Model 1, which is the reference model in this study, consists of two main layers of “hard” and “soft” cuticles. The hard cuticle in the model represents the epicuticle and the exocuticle layers in the real sample. The compliant endocuticle, which is the innermost layer in the vein multilayer structure, is referred here as the soft cuticle. This layer is connected to a resilin patch which is located in the contact area of the longitudinal and cross veins (Fig. 1B). Figure 2A shows Model 1 with the indication of its main components. The dimensions of the model are given in Fig. S1.

Model 2 was developed by replacing the soft endocuticle and the resilin patch in Model 1 by a layer of hard cuticle. The other characteristics of this model are totally similar to those of Model 1. Model 2 was used to investigate the effect of the removal of the soft endocuticle and the resilin on the mechanical behaviour of the joint. This model is shown in Fig. 2C.

The four-node bilinear plain strain elements with reduced integration (CPE4R) were employed for modelling the vein joint. The use of the reduced integration scheme considerably decreased the computational time required for the FE analysis. An enhanced hourglassing control was further utilized to avoid the possible mesh instability resulting from the use of the reduced integration method.

### Material properties

Wing veins in Odonata have been shown to have a complex lamellar structure composed of up to six cuticular layers^13^. However, as mentioned earlier, we simplified our model by considering two main layers of hard and soft cuticles. The hard cuticle in our model corresponds to a layer of epicuticle, two layers of exocuticle and probably one layer of mesocuticle. Therefore, in order to mitigate the effect of such a simplification we assigned an equivalent Young’s modulus of 3 GPa to the hard cuticle layer in our model. This value corresponds to the elastic modulus of fresh insect cuticle^18^. The resilin patch and the resilin-dominated endocuticle were assumed to have a Young’s modulus of 2 MPa^19^,^20^. The soft and the hard cuticle layers were considered to have a density of 1200 kg.m^−3^ 20 and 1000 kg.m^−3^ 18, respectively. The Poisson’s ratio of all parts was taken as 0.49^15^.

### Loadings and boundary conditions

The mechanical behaviour of the models was simulated under two loading conditions. We first applied the same point force to the free end of the cross veins in both models. The force was applied to both dorsal and ventral sides of the cross vein (Fig. 2A,B). This loading condition enabled us to assess the effect of the soft cuticle and the resilin patch on the deformability of the joint under external forces applied in flight. On the other hand, in order to analyse the influence of the mentioned structural elements on the mechanical behaviour of the joint in contact with an obstacle, we applied the same external displacement to the models. This displacement was applied to the ventral side of the cross vein at its free end (Fig. 2D,E). In both loading scenarios, the displacements of the longitudinal vein were constrained in all directions.

In this study, we used a nonlinear dynamic implicit analysis. The consideration of the nonlinear analysis allowed us to capture the effect of the changes in the geometric configuration of the joint due to large deformations.

### Data Availability

All supporting data are made available either in the article or the electronic supplementary material. The FE models can be made available on request: please contact HR at hrajabi@zoologie.uni-kiel.de.

## Additional Information

**How to cite this article**: Rajabi, H. *et al*. Resilin microjoints: a smart design strategy to avoid failure in dragonfly wings. *Sci. Rep.* **6**, 39039; doi: 10.1038/srep39039 (2016).

**Publisher's note:** Springer Nature remains neutral with regard to jurisdictional claims in published maps and institutional affiliations.

## Supplementary Material

Supplementary Information

Supplementary Video S1

Supplementary Video S2

Supplementary Video S3

Supplementary Video S4

Supplementary Video S5

Supplementary Video S6
