# Supplementary material for: Resilin microjoints: a smart design strategy to avoid failure in dragonfly wings
Source: Sci Rep. 2016 Dec 14;6:39039. doi: 10.1038/srep39039 (PMC5155300; doi:10.1038/srep39039)
Supplement: Supplementary Information [file srep39039-s1.pdf]

Resilin microjoints: a smart design strategy to avoid failure in dragonfly wings

Rajabi, H.[1,2*], Shafiei, A.[2], Darvizeh A.[2], and Gorb, S.N.[1]

[1] Institute of Zoology, Functional Morphology and Biomechanics, Christian-Albrechts-University, Kiel, Germany
[2] Department of Mechanical Engineering, University of Guilan, Rasht, Iran

* Corresponding author: hrajabi@zoologie.uni-kiel.de

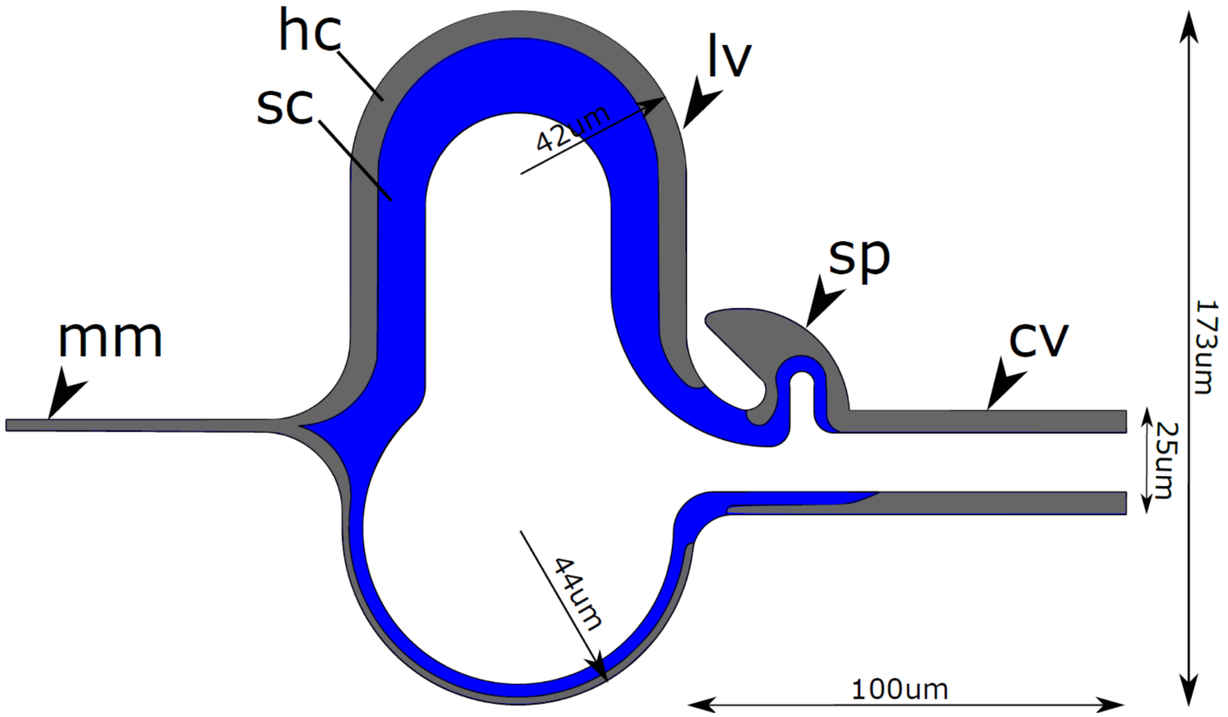

Fig. S1: Model 1 with the indication of the main components and dimensions. lv, longitudinal vein; cv, cross vein; mm, membrane; sp, spike; hc, hard cuticle; sc, softcuticle.

Video S1: A male dragonfly *Sympetrum sanguineum* trying to grip the female by the head. Several contracts may occur between wings and bodies during this process.

Video S2: Deformation of Model 1 subjected to a force applied to the dorsal side of the cross vein at its free end.

Video S3: Deformation of Model 1 subjected to a force applied to the ventral side of the cross vein at its free end.

Video S4: Deformation of Model 2 subjected to a force applied to the dorsal side of the cross vein at its free end.

Video S5: Deformation of Model 1 subjected to a deformation applied to the ventral side of the cross vein at its free end.

Video S6: Deformation of Model 2 subjected to a deformation applied to the ventral side of the cross vein at its free end.
